# Botanical formulation HX110B ameliorates PPE-induced emphysema in mice via regulation of PPAR/RXR signaling pathway

**DOI:** 10.1371/journal.pone.0305911

**Published:** 2024-07-25

**Authors:** Soojin Lee, Chang Hyung Lee, Jungkyu Lee, Yoonseon Jeong, Jong-Hyung Park, In-Jeong Nam, Doo Suk Lee, Hyun Myung Lee, Soo-Yeon Ahn, Eujung Kim, Seungyeon Jeong, Seung-Shin Yu, Wonwoo Lee

**Affiliations:** R&D Center for Innovative Medicines, Helixmith Co., Ltd., Seoul, Korea; Tokyo University of Agriculture, JAPAN

## Abstract

Chronic obstructive pulmonary disease (COPD), an inflammatory lung disease, causes approximately 3 million deaths each year; however, its pathological mechanisms are not fully understood. In this study, we examined whether HX110B, a mixture of *Taraxacum officinale*, *Dioscorea batatas*, and *Schizonepeta tenuifolia* extracts, could suppress porcine pancreatic elastase (PPE)-induced emphysema in mice and its mechanism of action. The therapeutic efficacy of HX110B was tested using a PPE-induced emphysema mouse model and human bronchial epithelial cell line BEAS-2B. *In vivo* data showed that the alveolar wall and air space expansion damaged by PPE were improved by HX110B administration. HX110B also effectively suppresses the expression levels of pro-inflammatory mediators including IL-6, IL-1β, MIP-2, and iNOS, while stimulating the expression of lung protective factors such as IL-10, CC16, SP-D, and sRAGE. Moreover, HX110B improved the impaired OXPHOS subunit gene expression. *In vitro* analysis revealed that HX110B exerted its effects by activating the PPAR-RXR signaling pathways. Overall, our data demonstrated that HX110B could be a promising therapeutic option for COPD treatment.

## 1. Introduction

COPD is the third most serious cause of mortality, accounting for approximately 6% of total deaths worldwide [1]. COPD is a progressive and heterogeneous disease associated with excessive inflammatory responses of the lungs to injurious gases and particles [2]. It is characterized by the destruction of the gas-exchanging surface followed by airflow limitation, which is not completely reversible. The typical symptoms of COPD are cough, breathlessness, dyspnea, and increased sputum production [3, 4]. Bronchodilators and inhaled corticosteroids can provide some symptomatic relief temporarily; however, there is no treatment to stop disease exacerbation [5, 6]. Thus, there is a huge unmet medical need to treat the underlying disease progression.

Emphysema and chronic bronchitis are the representative clinical phenotypes of COPD with different pathogenesis [7]. Emphysema is characterized by destruction of the alveolar airways and is a representative manifestation of progressive COPD [8]. The main cause of emphysema has not been established. However, cigarette smoking is considered a major risk factor for emphysema, leading to immune cell recruitment and epithelial cell activation [9, 10]. Infiltrated alveolar macrophages and neutrophils cause an imbalance between protease and antiprotease activity, which can result in alveolar tissue disruption [11]. Furthermore, emphysema progression can be aggravated by chronic inflammatory responses and oxidative stress [12].

Interleukin-6 (IL-6), interleukin-1β (IL-1β), macrophage inflammatory protein-2 (MIP-2), and inducible nitric oxide synthase (iNOS) are well-known pro-inflammatory mediators associated with lung injury [13]. IL-6 has been reported to be a predictive pro-inflammatory biomarker for sepsis and acute lung injury. IL-1β, MIP-2, and IL-6 are key inflammatory cytokines that induce neutrophil infiltration and activation [14–17]. iNOS is expressed by macrophages, neutrophils, and epithelial cells, and its expression can be upregulated by cytokines such as IL-1β [18]. Additionally, induction of iNOS causes excessive NO production and leads to indiscriminate nitrosylation by NO compounds, which can disrupt mitochondrial metabolism and cell necrosis [19, 20].

Interleukin-10 (IL-10), clara (Club) cell protein-16 (CC16), surfactant protein-D (SP-D), and soluble receptor for advanced glycation end products (sRAGE) are involved in the regulation of lung inflammation. IL-10 is a key cytokine that suppresses proinflammatory responses by resolving neutrophilic lung inflammation and deactivating macrophages. In several animal studies, IL-10 has been shown to reduce subepithelial fibrosis and inhibit airway inflammation by regulating the immune system [21–24]. CC16, secreted by bronchiolar Clara cells, is a therapeutic protein for lung epithelial injury. CC16 attenuates oxidative stress and inflammatory responses by inhibiting phospholipase A2 [25–27]. In many studies, serum levels of CC16 were reduced in patients with lung injury caused by smoking and air pollution. Furthermore, the reduced levels of CC16 may indicate airflow limitation and asthma [26, 28, 29]. SP-D is a major regulator of the host defense system that contributes to immune and inflammatory regulation by modulating immune cell activity. SP-D is a pulmonary collagen synthesized in respiratory epithelial cells and is often increased under epithelial inflammation-related conditions [30, 31]. A recent study showed that SP-D protects against structural damage, oxidative stress, and fibrosis by suppressing alveolar macrophage activity [32]. sRAGE is a soluble form of RAGE expressed by alveolar type I cells [33]. sRAGE binds to the membrane of alveolar epithelial cells and is released into the alveolar space during pulmonary inflammation [34]. sRAGE has anti-inflammatory effects and is strongly associated with the progression of emphysema [35, 36].

Peroxisome proliferator-activated receptors (PPARs) are well-known immunomodulators that regulate gene transcription by binding to specific elements of the target gene. PPARs are involved in the cellular responses of resident and structural cells involved in inflammation and tissue remodeling in chronic lung diseases. Among PPARs, especially because PPARγ has antioxidant and anti-apoptotic properties, it has generally been considered a notable anti-inflammatory target for respiratory diseases. PPARγ is activated by forming heterodimers with the retinoid X receptors (RXRs), and the PPARγ/RXR heterodimer can hinder the expression of mRNA for IL-6 and TNF-α, while encouraging that for anti-inflammatory cytokines such as IL-10 [37]. A recent study on airway related diseases revealed that the expression of PPARγ mRNA and protein levels is downregulated in alveolar macrophages in patients with asthma, unlike in healthy people. This indicates that downregulation of PPARγ expression is associated with exacerbation of pulmonary disease [38, 39]. In a similar vein, it has been reported that agonists of PPAR and RXR have therapeutic effects on various pulmonary disease models, including emphysema and asthma [40–45]. Particularly, RXRγ agonists have been shown to have significant treatment effects on emphysema patients [46]. The therapeutic effect of the PPAR/RXR signaling pathway on respiratory diseases was previously reported to be mediated by IL-10 and CC16 [47, 48]. Furthermore, PPARγ is involved in regulating proliferator-activated receptor gamma co-activator-1 alpha (PGC1α), which is a widely known regulator of mitochondrial oxidative metabolism. The activation of PPARγ/PGC1α signaling stimulates mitochondrial biogenesis, and PPARγ contributes to rescuing mitochondrial function while reducing reactive oxygen species [49–52]. Additionally, PPAR agonists not only increase mitochondrial mass but also significantly improve oxidative phosphorylation system (OXPHOS) gene expression impairment that causes metabolic dysfunction [53].

Three plants, *Dioscorea batatas*, *Taraxacum officinale*, and *Schizonepeta tenuifolia*, were selected for our study based on their functions. Their properties described in traditional Korean medicine literature are as follows [54]: *Dioscorea batatas* has been widely used for nutritional and medicinal purposes with various effects. *Dioscorea batatas* is known to be effective in lung nourishing, cough relief, and various inflammatory diseases owing to its antioxidant and anti-inflammatory activities [55–57]. *Taraxacum officinale* is known to have therapeutic effects on various respiratory diseases, such as lung abscesses, upper respiratory tract infections, emphysema, bronchitis, and pneumonia [58–60]. Liu et al. (2010) showed that a water extract of *T*. *officinale* suppressed LPS-induced acute lung injury (ALI) by decreasing pro-inflammatory cytokines production and regulating oxidative stress-related responses. Medical practitioners in Asian countries such as Korea and China have utilized *S*. *tenuifolia* to treat respiratory infections, including colds, fevers, and sore throat [61, 62]. Recent studies suggest that *S*. *tenuifolia* has immunomodulatory activities and can inhibit pulmonary inflammatory responses via regulating the TLR4 signaling pathway [63].

In our previous study, we developed HX110B, an ethanol-extracted mixture of *D*. *batatas*, *T*. *officinale*, and *S*. *tenuifolia* [54]. We revealed that HX110B regulates Nrf2-HO-1 signaling pathway and consequently ameliorates LPS-induced ALI [54]. Here, our aim is to investigate the therapeutic effects of HX110B on emphysema in a PPE-induced mouse model.

## 2. Materials and methods

### 2.1. Cell culture and reagents

All reagents used for cell culture were purchased from Invitrogen (USA) unless otherwise specified. PPE, ketoprofen, GW6471, GSK3787, SP16832, and HX531 were purchased from Sigma-Aldrich (USA). BEAS-2B cell line (ATCC, USA) was cultured in complete RPMI1640 medium (supplemented with 10% fetal bovine serum, 100 U/mL penicillin, and 100 μg/mL streptomycin) and incubated at 37°C in a humidified atmosphere containing 5% (v/v) CO_2_ until confluent.

### 2.2. Preparation of HX110B extract

HX110B was obtained as previously mentioned [54]. *Dioscorea batatas*, *Taraxacum officinale* and *Schizonepeta tenuifolia* were purchased from Humanherb Co., Ltd. (Gyeongsan, Korea). Their species were determined through DNA sequencing analysis (miDNA Genome Research Institute, Kunsan, Korea). HX110B was prepared by mixing three dried herbs in a weight ratio of 1:1:1, and then extracting the mixture with 25% ethanol. The voucher specimens used in this study were kept at the herbarium of Helixmith Co., Ltd. (Seoul, Korea). Voucher Specimen No: S14161213 for *Dioscorea batatas*; F02170525 for *Taraxacum officinale*; H2719121 for *Schizonepeta tenuifolia*.

### 2.3. High-performance liquid chromatography (HPLC)

The chemical characteristics of HX110B were analyzed using HPLC photodiode array (Waters Alliance e2695 system; Waters, USA). HX110B extract was dissolved in 50% methanol and filtered using a 0.45 μm polyvinylidene fluoride (PVDF) filter before use. The detailed conditions of the HPLC analysis are provided in our previous study [54].

### 2.4. Evaluation of correlation coefficient

The correlation coefficient (R) is used to evaluate equivalence, indicating the correlation between each peak area established in the standard chemistry profile (Batch 1) and each corresponding peak area in the equivalent target chemistry profile (Batch 2 and 3). The correlation coefficient was calculated using the function of Pearson correlation coefficient calculation in Excel program (Microsoft, USA).

### 2.5. Experimental animals

All animals (C57BL/6, male, 7 weeks old, Raonbio Inc., Yongin, Korea) used in the experiment were cared for according to the standard guidelines set by the Institutional Animal Care and Use Committee of Helixmith Co., Ltd. All mice were quarantined and acclimatized by housing in an air-conditioned animal facility for at least 7 days under a 12-hour light and dark cycle. All experimental procedures were approved by the Institutional Animal Care and Use Committee of Helixmith Co., Ltd. (Approval Number: VIC-20-11-001).

### 2.6. PPE-induced emphysema mouse model

PPE was prepared by dissolving in saline (0.9% NaCl) at 1 unit/head. The mice were then randomly allocated into five groups (n = 6): NC, PPE, HX110B (50 mg/kg), HX110B (100 mg/kg), and HX110B (200 mg/kg). For administration, anesthesia was induced by inhalation using 2% isoflurane (Hana Pharm Co., Ltd, Korea) with a tabletop anesthesia system, and then the mice received a single intratracheal injection of either PPE (1 unit) or saline. Afterwards, all animals were injected intraperitoneally with ketoprofen (5mg/kg in saline) to alleviate pain, followed by daily oral administration of HX110B for 3 weeks. After treatment, the mice were sacrificed by CO_2_ inhalation and analyzed. For lung tissue harvesting, animals were anesthetized, exsanguinated, and euthanized immediately. Fresh paraformaldehyde (PFA) was immediately injected through the tracheal cannula at a constant rate until the lungs became taut. The lungs filled with PFA were maintained for at least 2 minutes without releasing the tied trachea in the maximally inflated state.

### 2.7. H&E staining

Lung samples fixed in 4% PFA were serially dehydrated with graded ethanol series, embedded in paraffin, and sectioned at 4 μm thickness. The sections were stained with hematoxylin and eosin (H&E). The air space area was calculated for each mouse by quantifying the number of intersections between the horizontal and vertical grid lines and alveolar walls in 10 non-overlapping fields.

### 2.8. RNA isolation and qRT-PCR analysis

Total RNA from mouse lungs and cell lines was extracted and purified using TRIzol (Invitrogen), and cDNA was synthesized using oligo dT primers (QIAGEN, USA) and Reverse Transcriptase XL (Takara, Japan) from 1 μg of RNA. Real-time quantitative RT-PCR was performed using SYBR Premix (Takara) and a Thermal Cycler Dice Real Time System TP800 (Takara). PCR was conducted with initial denaturation at 95°C for 5 s, annealing, and elongation at 60°C for 30 s. A list of sequences of the qPCR primer pairs is provided in S1 Table.

### 2.9. Luciferase reporter plasmid assay

The luciferase reporter assay was performed following a previously described method [54, 64, 65]. Briefly, BEAS-2B cells were transfected with a PPRE-reporter plasmid (QIAGEN) or RXRE-reporter plasmid (QIAGEN) using Lipofectamine 3000 (Invitrogen). After 24 h, transfected cells were treated with various concentrations of HX110B and receptor-specific antagonists for 18 h. The cell lysates were extracted and used for luciferase activity assays using a dual luciferase reporter assay system (Promega, USA) and Varioskan LUX (Thermo Fisher, USA), following the manufacturer’s instructions.

### 2.10. Statistical analysis

Three independent experiments were conducted, and all values are expressed as the mean ± SEM. Student’s t-test was employed to statistically analyze the differences between two groups, while one-way analysis of variance (ANOVA) with Tukey’s correction was utilized for conducting multiple comparisons.

## 3. Results

### 3.1. Quality of HX110B is controlled by HPLC analysis

**Fig 1:**
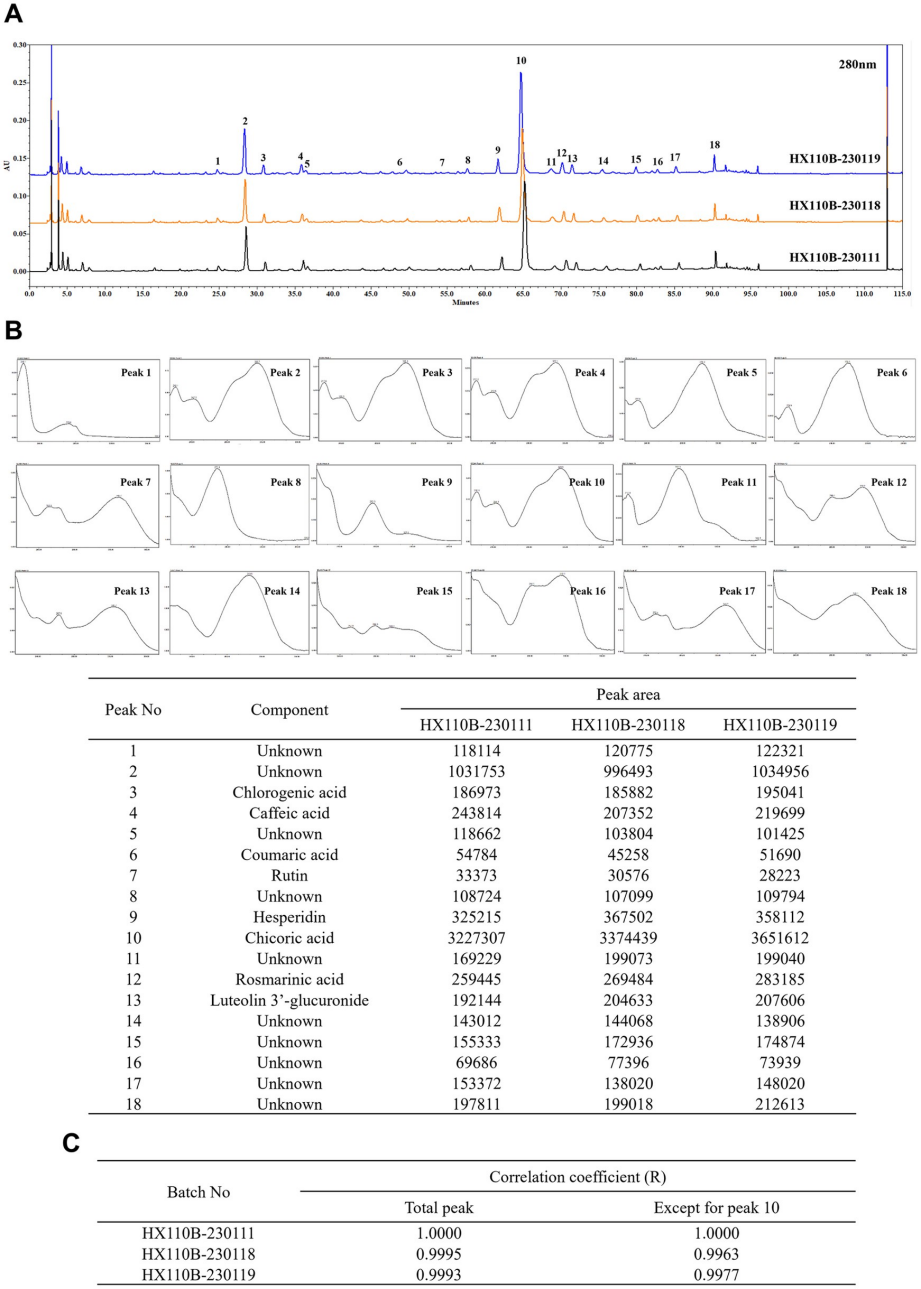
Details of the quality control of HX110B. The batch-to-batch consistency of HX110B was ensured by monitoring its HPLC profile. (A) HPLC profiles of HX110B extract. (B) UV spectra of the peak and measured peak area. (C) Calculated Correalation coefficient between the three different batches of HX110B.

The results of HPLC profiling of HX110B are shown in Fig 1A. To confirm batch-to-batch consistency, a total of 18 peaks were selected as representative components, and the UV spectra of these peaks were analyzed to identify the major components contained in HX110B (Fig 1B). Equivalence between the three HX110B batches was evaluated based on the selected peaks of batch 1. As a result, each equivalence showed a significantly high correlation of over 0.999 (Fig 1C). In addition, since equivalence is affected by the peak area, equivalence was evaluated except for peak 10, which occupies about 50% of the area. Even in that case, there was a high equivalence of 0.990 or more between the three different batches. Taken together, it was found that the quality of raw materials produced through the manufacturing process of HX110B was kept constant.

### 3.2. HX110B ameliorates PPE-induced lung emphysema

**Fig 2:**
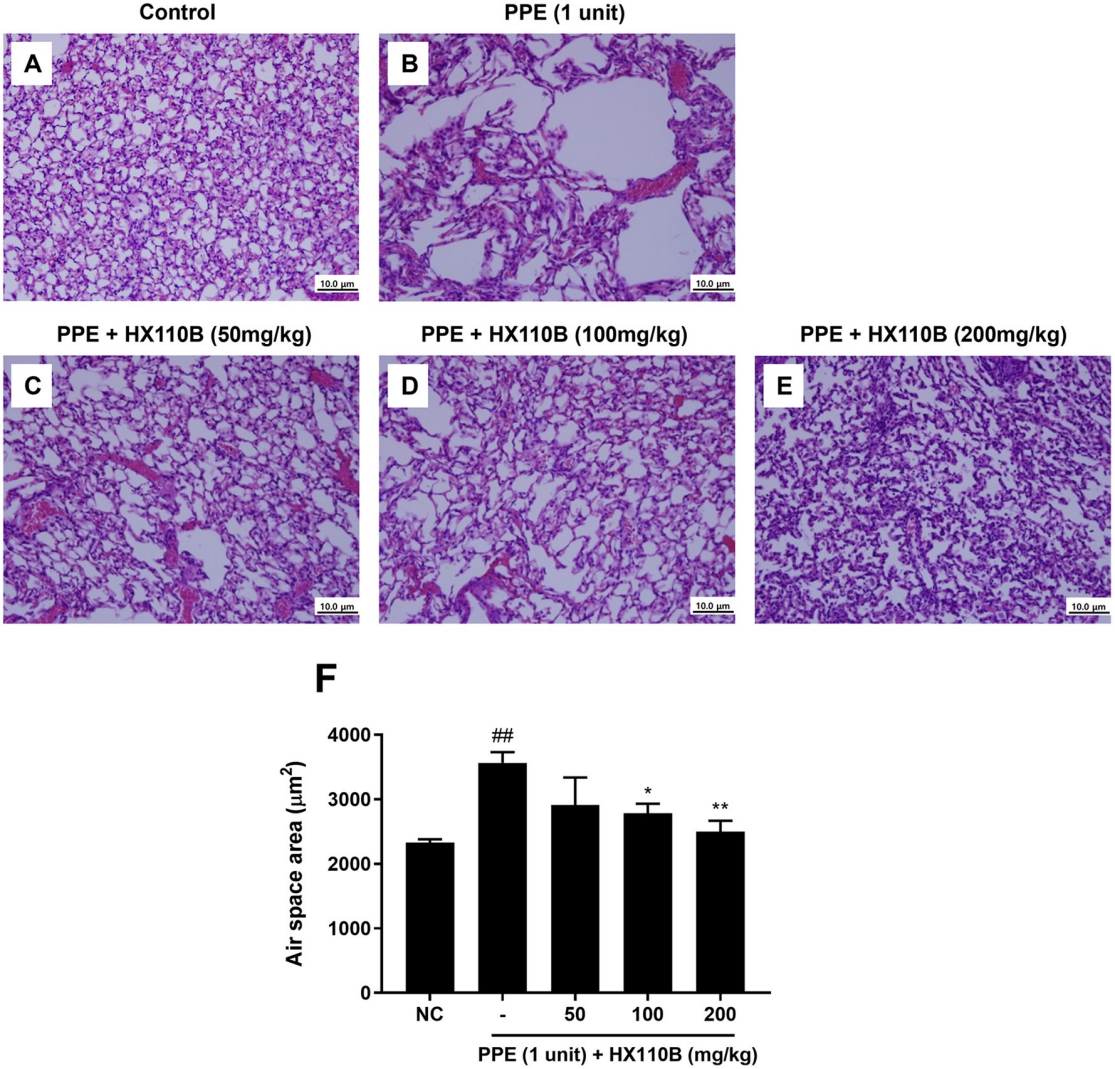
Effects of HX110B on PPE-induced emphysema. The ameliorating effects of HX110B on PPE-induced emphysema were examined as described in the Materials and Methods section. (A-E) Histological images of H&E-stained lung tissue sections. (F) Quantification of the pulmonary air space area. ##p < 0.01 vs. the NC group, *p < 0.05, **p < 0.01 vs. the PPE group.

In our previous research, we developed HX110B composed of three plants that produce synergistic effects on inflammatory responses [54]; therefore, we hypothesized that HX110B would have therapeutic effects on emphysema, a respiratory inflammation disease. The therapeutic effects of HX110B on emphysema-like conditions were evaluated using a mouse emphysema model induced by PPE. Mice were administered 1 unit of PPE intratracheally and orally administered three different doses of HX110B (50, 100, and 200 mg/kg) daily for 3 weeks. Representative results from the histological assessment are shown in Fig 2. H&E staining was used to estimate the changes in lung structure induced by HX110B at different concentrations. As shown in Fig 2A–2E, PPE-treated mice showed a destroyed lung architecture with enlargement of the airspace, whereas HX110B-treated mice showed significantly improved PPE-induced alveolar wall damage in a dose-dependent manner. Quantitative analysis of the air space area also indicated that HX110B treatment attenuated the exacerbation of air space enlargement mediated by PPE (Fig 2F).

### 3.3. HX110B reduces the mRNA level of pro-inflammatory mediators in the lung of PPE-treated mice

**Fig 3:**
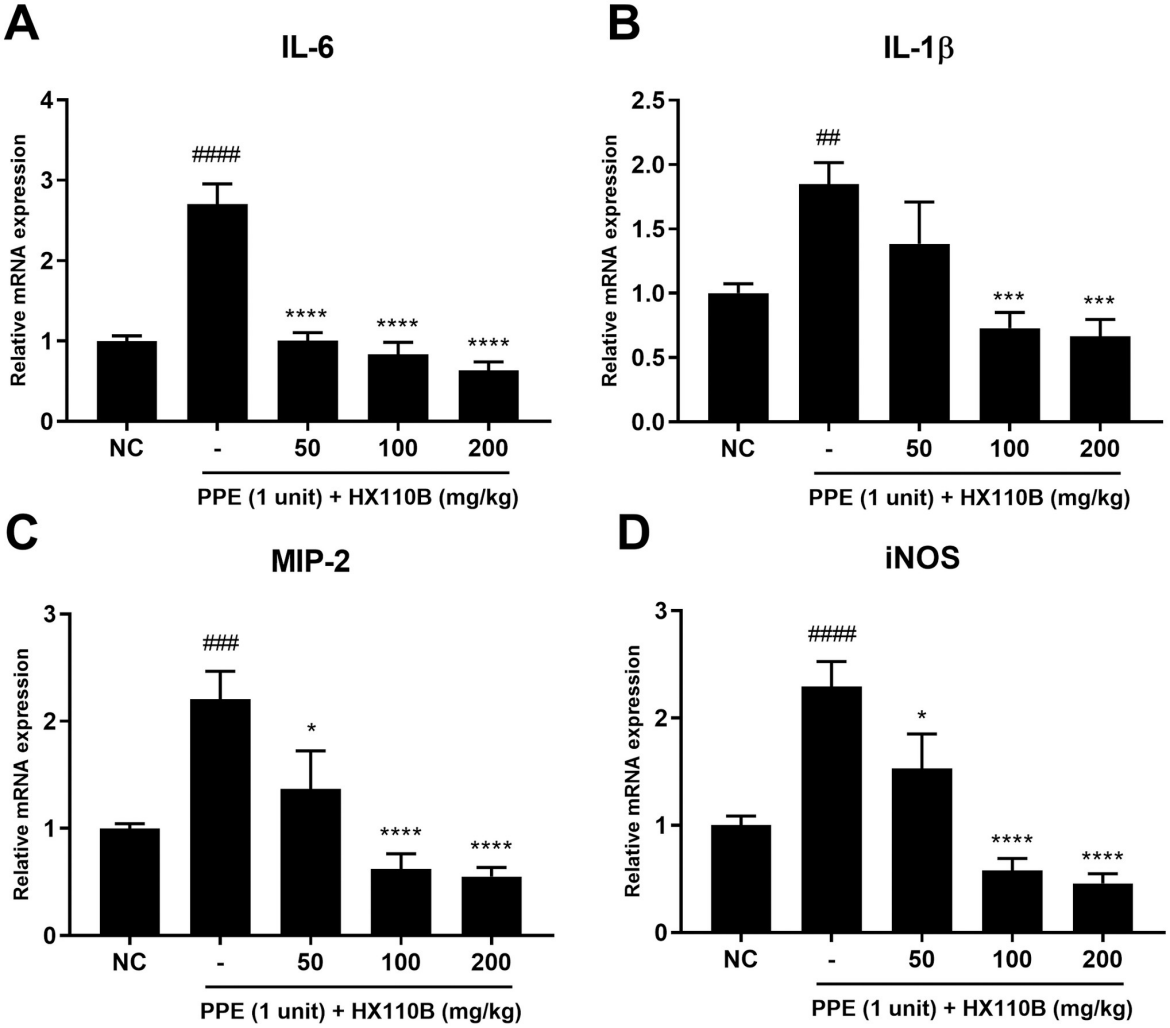
Effects of HX110B on the expression of pro-inflammatory mediators in mice lungs. Total RNA was extracted from mouse lungs and subjected to analysis for IL-6, IL-1β, MIP-2, and iNOS using qRT-PCR. (A-D) Changes in the RNA level of pro-inflammatory mediators. ##p < 0.01, ####p < 0.0001 vs. the NC group; **p < 0.01, ***p < 0.001, ****p < 0.0001 vs. the PPE group.

IL-6, IL-1β, MIP-2, and iNOS are well known to be important for the development and worsening of lung disease [66–69]. As shown in Fig 3, the mRNA expression levels of IL-6, IL-1β, MIP-2, and iNOS were intensely increased in the PPE-treated group, but those of HX110B-treated groups were significantly diminished. These results showed that HX110B may help alleviate the worsening of lung injury by controlling the production of pro-inflammatory mediators.

### 3.4. HX110B improves the diminished expression of lung-protective factor in the lung of PPE-treated mice

**Fig 4:**
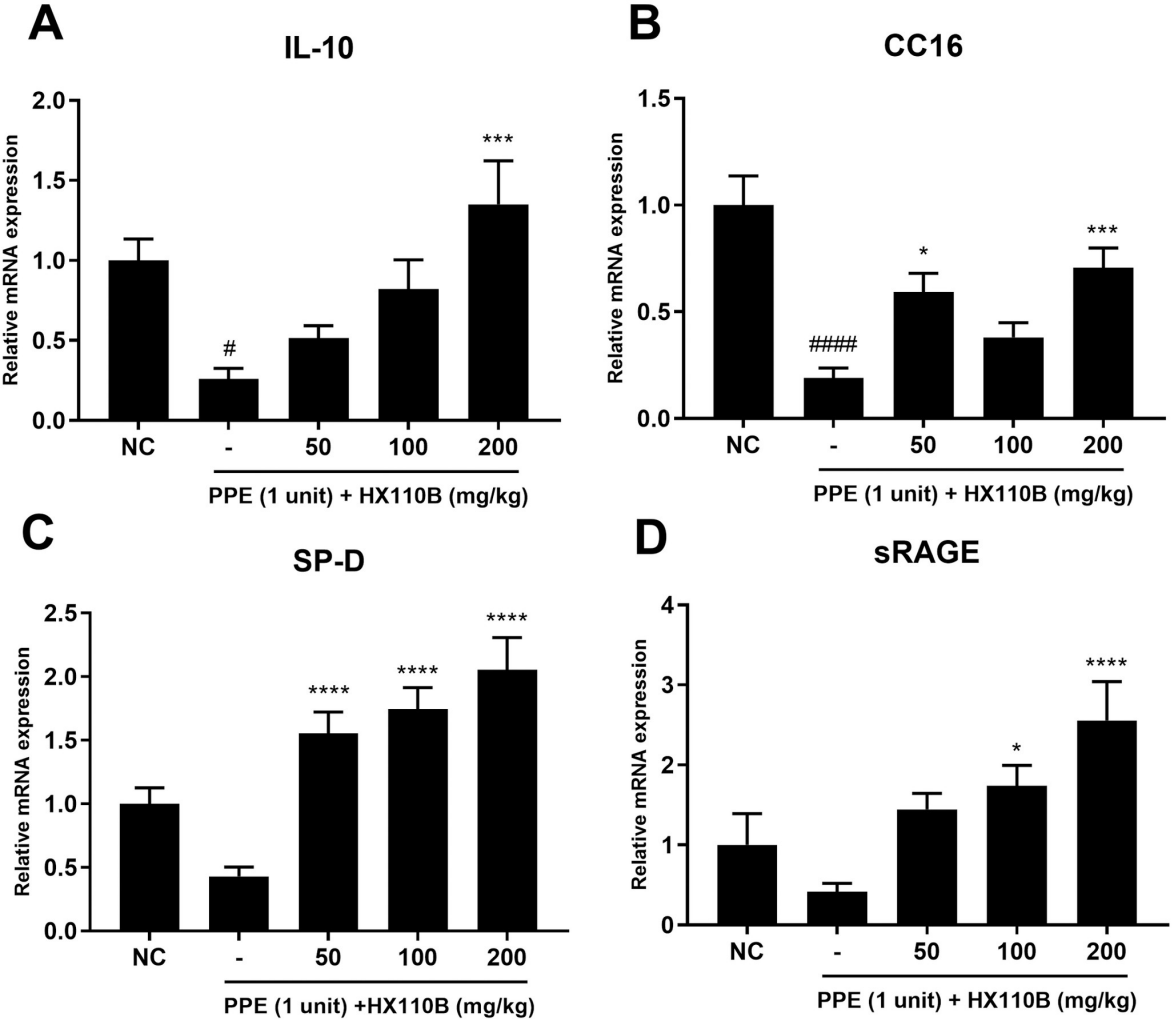
Effects of HX110B on the expression of lung-protective factors in mice lungs. Total RNA extracted from mouse lungs was analyzed for IL-10, CC-16, SP-D, and sRAGE using qRT-PCR. (A-D) Changes in the RNA level of lung-protective factors in mice lungs were measured. ###p < 0.001, ####p < 0.0001 vs. the NC group; *p < 0.05, **p < 0.01, ***p < 0.001, ****p < 0.0001 vs. the PPE group.

The lung-protective effect of HX110B was further tested by determining the expression levels of IL-10, CC16, SP-D, and sRAGE, which have been reported to ameliorate the pathological manifestations of emphysema. These factors are known to suppress pro-inflammatory responses in lung tissues. In particular, CC16 and SP-D have been reported to possess lung-protective properties against oxidative stress, which can lead to the deterioration of emphysema [25–27, 32]. In the elastase-treated condition, the RNA expression levels of these factors were greatly reduced compared to those in the NC group; however, these effects were significantly improved when the mice were administered HX110B (Fig 4).

### 3.5. HX110B improves the expression of OXPHOS genes in the lung of PPE-treated mice

**Fig 5:**
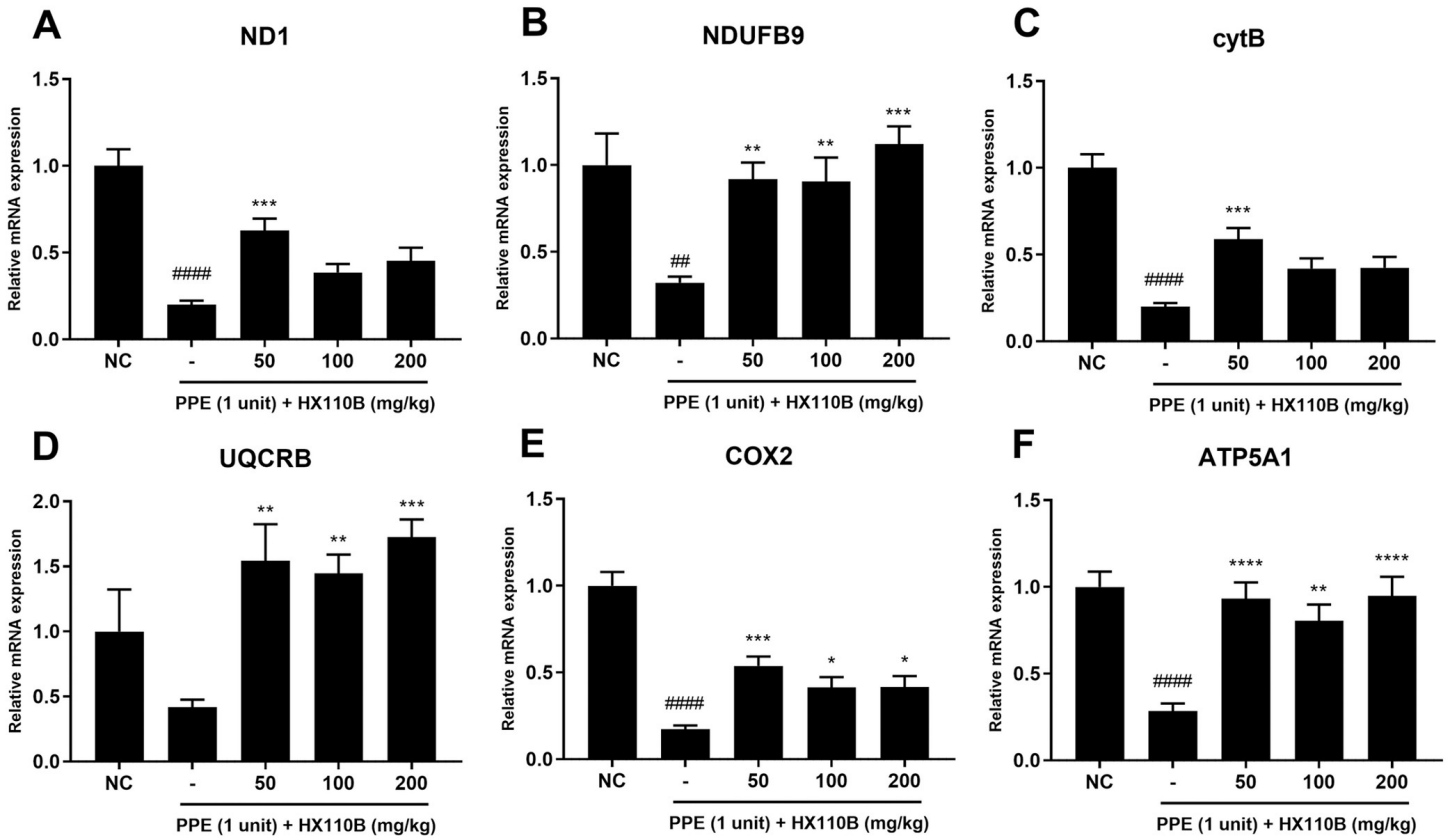
Effects of HX110B on the expression of OXPHOS genes in mice lungs. Total RNA extracted from mouse lungs was analyzed for ND1, NDUFB9, CytB, UQCRB, COX2, and ATP5A1 using qRT-PCR. (A-F) Changes in the RNA level of OXPHOS genes in mice lungs were measured. ###p < 0.001, ####p < 0.0001 vs. the NC group; *p < 0.05, **p < 0.01, ***p < 0.001, ****p < 0.0001 vs. the PPE group.

Recent studies have demonstrated that dysfunction of mitochondria is related to COPD pathophysiology [70]. Metabolic dysfunctions, which are mainly mediated by impaired OXPHOS gene expression, are found in patients with COPD because they contribute to exacerbating lung disease conditions [70, 71]. In this context, OXPHOS gene expression was evaluated to verify the potential therapeutic efficacy of HX110B in improving mitochondrial dysfunction-related gene expression. Fig 5 shows the changes in the expression levels of OXPHOS subunit genes, including ND1 and NDUFB9 for complex I, CytB and UQCRB for complex III, COX2 for complex IV, and ATP5A1 for complex V. The RNA levels of OXPHOS subunits were markedly reduced by PPE treatment; however, the administration of HX110B significantly improved the expression levels of these factors (Fig 5).

### 3.6. HX110B upregulates the expression of IL-10 and CC16 in BEAS-2B cells

**Fig 6:**
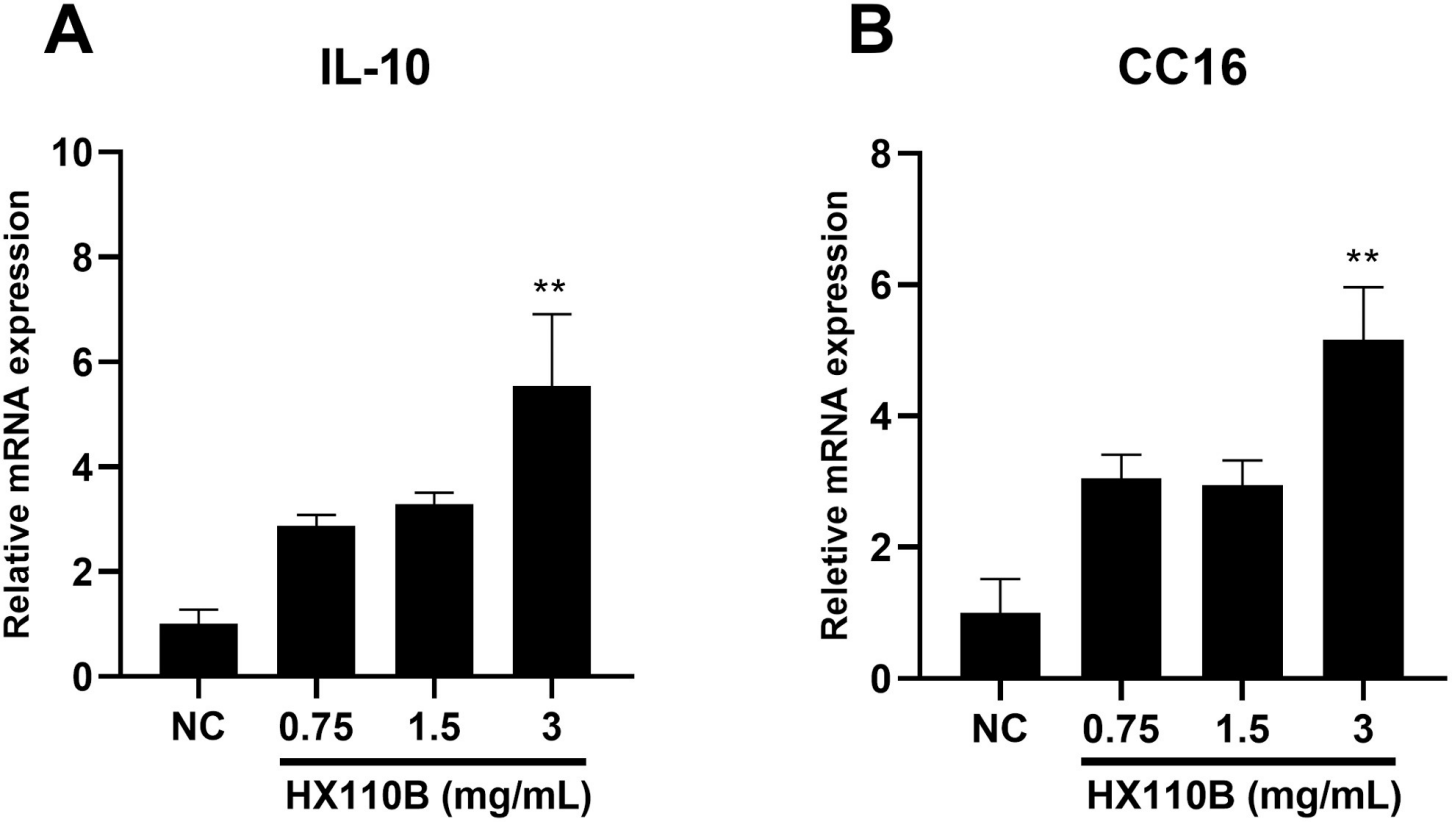
Effects of HX110B on the expression of IL-10 and CC16 in BEAS-2B cells. BEAS-2B cells were cultured with various concentrations of HX110B (0.75, 1.5, and 3 mg/mL) for 48 h. Total RNA was extracted and analyzed for IL-10 and CC16 using qRT-PCR. (A and B) Changes in IL-10 and CC16 RNA levels were measured. **p < 0.01 vs. the NC group.

To identify the detailed mechanism of HX110B based on in vivo studies, the BEAS-2B derived from normal human bronchial epithelium was used to evaluate the changes in lung-protective factors with and without HX110B treatment. HX110B was used to treat cells at concentrations of 0.75, 1.5, and 3.0 mg/mL for 48 h, and the expression levels of IL-10 and CC16 were quantified by measuring the mRNA levels. Both mRNA expression levels of IL-10 and CC16 were relatively increased in the HX110B-treated group at all doses in comparison with those in the NC group; in particular, the 3.0 mg/mL treatment group showed a prominent increase in expression (Fig 6). However, the expression of SP-D and sRAGE did not change after HX110B treatment (S1 Fig).

### 3.7. PPAR and RXR signaling pathways play a prominent role in the induction of IL-10 and CC16 gene expression by HX110B

**Fig 7:**
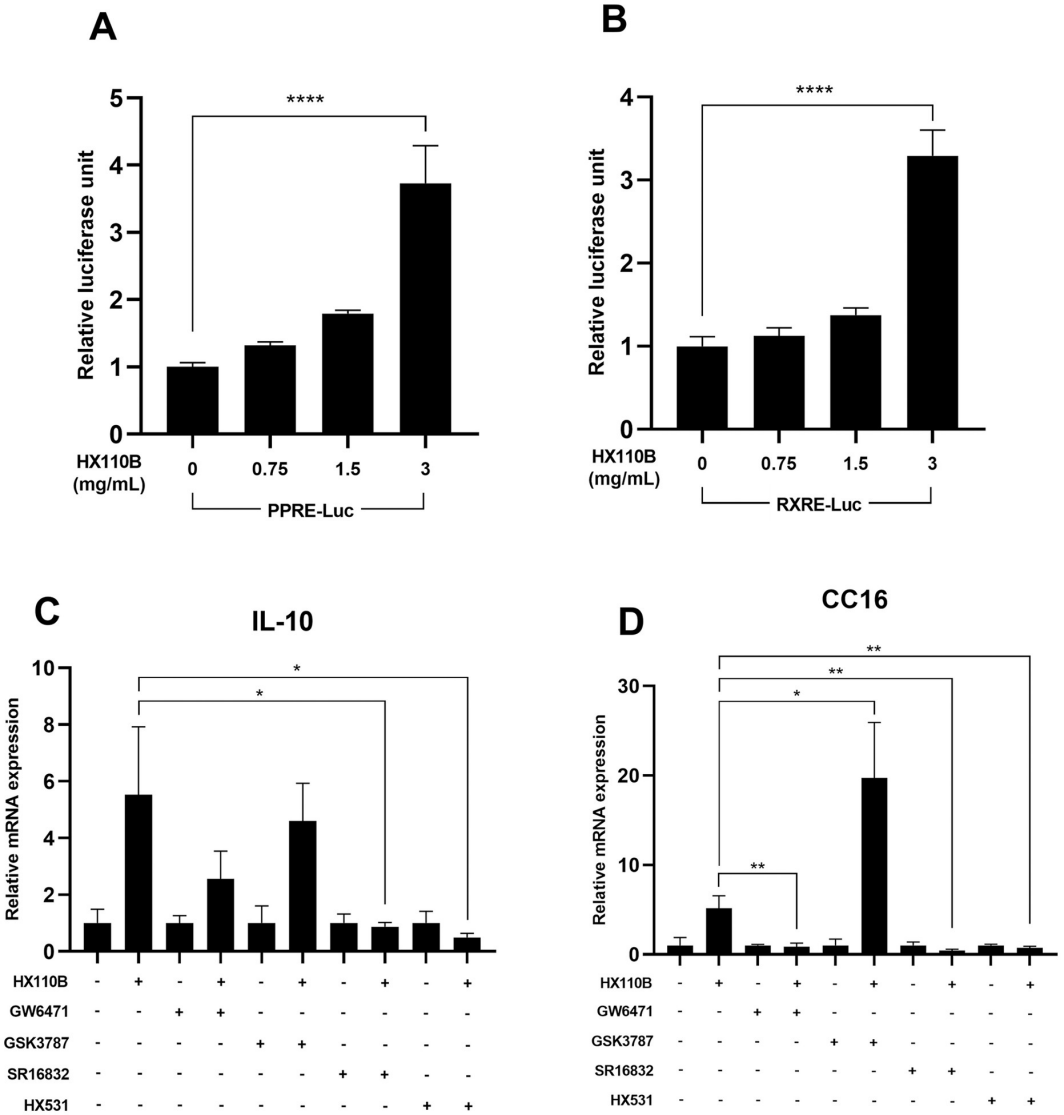
HX110B regulates PPAR and RXR signaling pathways in BEAS-2B cells. The effects of HX110B on PPAR and RXR signaling pathways were examined using luciferase activity assay and quantitative RT-PCR. The experimental protocol is detailed in the Materials and Methods section. (A and B) Changes in luciferase activity. (C and D) Changes in the RNA levels of IL-10 and CC16 were. *p < 0.05, **p < 0.01, ***p < 0.001, ****p < 0.0001.

Because PPAR and RXR are well-known regulators of inflammation-related gene expression [37], we used a luciferase reporter plasmid containing PPRE or RXRE sequences to investigate whether HX110B regulates mitochondrial function and ROS-related factors through the PPAR-RXR signaling pathway. As a result, when the cells transfected with PPRE- or RXRE-containing plasmids were treated with HX110B, the luciferase activity was enhanced as the concentration of HX110B increased; in particular, the HX110B-treated group at 3 mg/mL demonstrated superior results compared to those from other concentrations (Fig 7A and 7B).

To further verify the relationship between PPARs and RXR, the following receptor-specific antagonists were used: PPARα (GW6471), PPARβ (GSK3787), PPARγ (SP16832), and RXR (HX531). When cells were treated with each antagonist, the gene expression levels of both IL-10 and CC16 were significantly reduced when cells were co-treated with GW6471, SP16832, or HX531. Interestingly, in the case of CC16, it increased when cells were co-treated with a PPARβ antagonist (Fig 7C and 7D). Overall, these data suggest that HX110B may regulate the expression of IL-10 and CC16 through the PPARα, PPARγ, and RXR signaling pathways.

## 4. Discussion

In this study, we prepared HX110B, consisting of three herbal plants: *T*. *officinale*, *D*. *batatas*, and *S*. *tenuifolia*. We used a PPE-induced mouse model and an in vitro model using BEAS-2B cells to determine the therapeutic effects of HX110B on emphysema. The results demonstrated that HX110B treatment improved the PPE-induced respiratory symptoms and pathological characteristics in vivo. HX110B treatment promotes the gene expression of lung-protective factors such as IL-10, CC16, SP-D, and sRAGE, and upregulates the expression of OXPHOS genes that are closely related to mitochondrial regulation, leading to a decrease in pro-inflammatory mediators. In vitro analysis revealed that HX110B exerted its effects by activating the PPAR-RXR signaling pathways. Overall, our data indicate that HX110B may be an efficacious therapeutic agent for emphysema.

The lungs have the largest internal surface area of approximately 150 m^2^. The lungs are continually exposed to the external environment with the largest surface area, which is highly efficient for the uptake and transfer of oxygen to tissues. Therefore, it is vulnerable to exogenous pollutants, contributing to increased oxidative stress and the generation of free radicals from inflammatory cells [72, 73]. The lung has an effective and highly specialized antioxidant defense mechanism with various antioxidants, including glutathione, vitamins, superoxide dismutase, and enzymes regulated by a redox-sensitive Nrf2 transcription factor to maintain homeostasis [73–76]. However, oxidative stress, which can lead to DNA, lipid, and protein damage, is induced when an imbalance occurs between antioxidants and oxidants [77]. Increased oxidative stress has a very close correlation with the pathophysiology of various lung diseases, for instance, ARDS, COPD, and idiopathic pulmonary fibrosis (IPF). Oxidative stress is one of the major factors contributing to the progression of lung disease; for example, both COPD and IPF patients exhibit increased oxidative stress levels as well as decreased antioxidant defenses, when compared with normal individuals [78, 79]. In our previous study, we revealed that HX110B markedly suppresses oxidative stress responses via the Nrf2-HO-1 signaling pathway. Therefore, it is possible that the therapeutic effects of HX110B on PPE-induced emphysema may be due to antioxidative stress.

Diverse botanical extracts and their marker compounds have been reported to contribute to the suppression of excessive inflammation [80, 81]. Allantoin, chicoric acid, and luteolin-3’-glucuronide are effective marker compounds of HX110B involved in the regulation of inflammatory responses. Allantoin is a natural and non-toxic compound that is efficacious against lung inflammation and oxidative stress [82, 83]. Furthermore, allantoin attenuates oxidative stress via the SIRT1/Nrf2 pathway in the MCD-induced NASH mouse model [84]. Chicoric acid (CA) is a promising natural antioxidant with various biological activities, including anti-inflammatory properties. Recent studies have revealed that CA restrains IL-1β, IL-6, and TNF-α and downregulates the NF-κB pathway, thereby diminishing oxidative stress and the inflammatory response [85–87]. Furthermore, CA also upregulates Nrf2, HO-1, PPARγ, PGC-1α, and SIRT1 expression and ameliorates mitochondrial function through the PI3K/AKT pathway [87–89]. Luteolin possesses the properties for anti-inflammatory, antioxidant, and antiviral effects. Luteolin-3’-glucuronide, a major metabolite in systemic circulation, was shown to influence the inflammatory response by encouraging the deactivation of transcription factors such as NF-κB and AP-1 [90–92]. Consequently, the anti-inflammatory effects of HX110B might be due to the synergistic effects of the properties of the respective components in HX110B.

Mitochondria are considered potential targets for the treatment of lung diseases because they play a critical role in lung function by regulating cellular metabolism and airway immune responses. As mitochondria participate in cellular process regulation, for example cell signaling and cell death, mitochondrial dysfunction may lead to airway contractility, oxidative stress, and apoptosis, which are implicated in the pathological progression of lung diseases including COPD, IPF, and asthma [70, 93–95]. More than 40 different cell types composing the lung depend on mitochondrial metabolism, and among them, cytotoxic exposure of lung epithelial cells reduces mitochondrial gene expression and OXPHOS activity. Additionally, OXPHOS gene expression is abnormally regulated in human bronchial smooth muscle cells of COPD patients, resulting in a decreased mitochondrial number and increased cyclooxygenase activity [95, 96]. Similarly, in another study, a decrease in OXPHOS gene expression was observed in lung tissue and alveolar type 2 cells of patients with emphysema, resulting in a decrease in the number of mitochondria [71]. In this study, HX110B restored OXPHOS gene expression to normal levels in emphysema-like conditions. This indicates that HX110B can improve mitochondrial dysfunction that causes various respiratory diseases by regulating OXPHOS activity. To verify this possibility, we are currently determining the effect of HX110B on mitochondrial function using various techniques, such as measuring mitochondrial mass, mitochondrial membrane potential value, and oxygen consumption rate.

IL-10 is a representative immunoregulatory cytokine known to suppress the development of COPD by regulating the inflammatory response in lung tissue [97–99]. Similarly, it has been reported that COPD patients have lower IL-10 concentrations in their blood [100, 101]. Additionally, CC-16 is a factor that aids in the recovery of lung injury, and decreased expression levels are observed in patients with lung damage due to smoking [27]. Moreover, it has been reported that CC-16-deficient mice exhibit COPD-like symptoms [102]. Consequently, IL-10 and CC-16 are very important factors that regulate the development of emphysema. Furthermore, they are not limited to emphysema, as they have been reported to contribute to various human diseases. For instance, the precise function of CC16 is not clearly understood; however, a recent study suggested that CC16 could be a target gene for lung cancer treatment, as it has immunomodulatory, anti-inflammatory, and anti-cancer effects [103]. Furthermore, serum CC16 concentrations are closely related to the severity of cystic fibrosis owing to its effect on systemic inflammation [104]. IL-10, a well-known anti-inflammatory cytokine, has been reported to have therapeutic effects in various inflammatory diseases such as autoimmune disease, tissue damage, cancer, chronic renal disease, and inflammatory bowel disease [105, 106]. It is also known that IL-10 exhibits therapeutic effects in various fibrotic diseases, including IPF and nonalcoholic steatohepatitis, by inhibiting the pro-fibrotic signaling pathway [107, 108]. HX110B significantly enhanced the expression levels of CC16 and IL-10 via the PPAR and RXR signaling pathways. Moreover, because the PPAR-RXR system exists throughout the body, HX110B has the potential to exhibit therapeutic effects in diverse diseases, including COPD.

As shown in the HPLC profile in Fig 1, HX110B contains a variety of polyphenols and flavonoid compounds. Which of these active compounds mediates the efficacy of HX110B has not yet been determined. Similar to the unique properties of botanical extracts, in which various ingredients simultaneously modulate various targets, the effect of HX110B is likely to be a combination of the effects of several constituents, rather than a single specific compound. Among the components included in HX110B, those related to the PPAR activation mechanism of HX110B identified in this study are as follows. Chicoric acid, which is contained in the highest amount in HX110B, has been shown to treat methotrexate-induced liver injury by activating the PPARγ signaling pathway [89]. Chlorogenic acid significantly increased mRNA and protein expression of PPARγ in the 3T3-L1 preadipocyte cell line [109]. Anti-depressant-like and anti-inflammatory effects of rosmarinic acid have been reported to be inhibited by PPARγ antagonists [110, 111]. Hesperidin exhibits anti-apoptotic effects through activation of PPARγ, thereby ameliorating ischemia and reperfusion injury [112]. Therefore, various components included in HX110B are expected to act comprehensively to activate the PPAR signaling pathway efficiently. To accurately identify this possibility, we are conducting molecular docking simulation study to calculate the binding modes and binding free energies between the PPAR and each component.

A limitation of this study is that the expression of all genes was tested only at the mRNA level. Since all gene expressions must be translated into protein to be effective, testing only changes in mRNA expression cannot verify whether the gene is functionally active. However, in this study, there were several technical problems, and it was difficult to measure protein expression under our experimental conditions. First, all available ELISA kits were of a grade that did not guarantee animal tissue sampling. In fact, when ELISA was performed on the whole extract of lung tissue using these kits, a significant level of background signal that interfered with the normal data analysis was observed. The main cause of this phenomenon is probably the matrix effect that commonly occurs during ELISA analysis of animal tissue samples. Moreover, excessive background signals were also observed when performing western blotting and immunohistochemical analysis, which is expected to be due to the low specificity of the primary antibody used. Due to these technical issues, in this study, it was inevitable to indirectly confirm the effect of HX110B by measuring mRNA expression levels. However, as shown in Fig 2, lung damage caused by PPE was restored to a normal level by HX110B administration, and similar gene expression profile changes were observed by HX110B treatment in other lung disease model experiments ([54] and unpublished data). Therefore, we believe that the HX110B-induced RNA expression changes were responsible for the therapeutic effect of HX110B. We plan to introduce a sensitive protein analysis technique, such as the TR-FRET assay, to identify the precise mechanism of action underlying the therapeutic effects of HX110B.

In conclusion, HX110B has high potential for development as a therapeutic agent for COPD due to its multiple mechanisms of action that were identified in this study. The plants used in HX110B formulation have been used by humans for a long time and are therefore considered safe. Indeed, HX110B did not show any adverse effects in the acute- or repeated-dose toxicity studies. To verify the potential of HX110B as a treatment for COPD, we are currently planning a clinical trial involving individuals who complain of abnormal respiratory symptoms.

## Supporting information

S1 Table Primer sequences. (DOCX)

S1 Fig TADIOS does not significantly regulate the expression of SP-D and sRAGE in BEAS-2B cells. (DOCX)

## References

[1] Lawless M, Burgess M, Bourke S. Impact of COVID-19 on Hospital Admissions for COPD Exacerbation: Lessons for Future Care. Medicina (Kaunas). 2022;58: 66. doi: 10.3390/medicina58010066 35056374 PMC8778793

[2] Brusselle GG, Joos GF, Bracke KR. New insights into the immunology of chronic obstructive pulmonary disease. Lancet. 2011;378: 1015–1026. doi: 10.1016/S0140-6736(11)60988-4 21907865

[3] Miravitlles M, Worth H, Soler Cataluña JJ, Price D, De Benedetto F, Roche N, et al. Observational study to characterise 24-hour COPD symptoms and their relationship with patient-reported outcomes: results from the ASSESS study. Respiratory Research. 2014;15: 122. doi: 10.1186/s12931-014-0122-1 25331383 PMC4220061

[4] Miravitlles M, Ribera A. Understanding the impact of symptoms on the burden of COPD. Respiratory Research. 2017;18: 67. doi: 10.1186/s12931-017-0548-3 28431503 PMC5399825

[5] Courtney J-M, Spafford PL. The Role of Epithelial-Mesenchymal Transition in Chronic Obstructive Pulmonary Disease. Cells Tissues Organs. 2017;203: 99–104. doi: 10.1159/000450919 28214877

[6] Czira A, Turner M, Martin A, Hinds D, Birch H, Gardiner F, et al. A systematic literature review of burden of illness in adults with uncontrolled moderate/severe asthma. Respir Med. 2022;191: 106670. doi: 10.1016/j.rmed.2021.106670 34883444

[7] Liu H, Tang H-Y, Xu J-Y, Pang Z-G. Small airway immunoglobulin A profile in emphysema-predominant chronic obstructive pulmonary disease. Chin Med J (Engl). 2020;133: 1915–1921. doi: 10.1097/CM9.0000000000000863 32826454 PMC7462224

[8] Vogelmeier CF, Criner GJ, Martinez FJ, Anzueto A, Barnes PJ, Bourbeau J, et al. Global Strategy for the Diagnosis, Management, and Prevention of Chronic Obstructive Lung Disease 2017 Report: GOLD Executive Summary. Eur Respir J. 2017;49: 1700214. doi: 10.1183/13993003.00214-2017 28182564

[9] Khedoe PPSJ, Wong MC, Wagenaar GTM, Plomp JJ, van Eck M, Havekes LM, et al. The Effect of PPE-Induced Emphysema and Chronic LPS-Induced Pulmonary Inflammation on Atherosclerosis Development in APOE*3-LEIDEN Mice. PLOS ONE. 2013;8: e80196. doi: 10.1371/journal.pone.0080196 24303000 PMC3841138

[10] Boschetto P, Quintavalle S, Miotto D, Lo Cascio N, Zeni E, Mapp CE. Chronic obstructive pulmonary disease (COPD) and occupational exposures. J Occup Med Toxicol. 2006;1: 11. doi: 10.1186/1745-6673-1-11 16756686 PMC1513231

[11] Barnes PJ. Chronic obstructive pulmonary disease. N Engl J Med. 2000;343: 269–280. doi: 10.1056/NEJM200007273430407 10911010

[12] Sharafkhaneh A, Hanania NA, Kim V. Pathogenesis of emphysema: from the bench to the bedside. Proc Am Thorac Soc. 2008;5: 475–477. doi: 10.1513/pats.200708-126ET 18453358 PMC2645322

[13] Polverino E, Rosales-Mayor E, Dale GE, Dembowsky K, Torres A. The Role of Neutrophil Elastase Inhibitors in Lung Diseases. Chest. 2017;152: 249–262. doi: 10.1016/j.chest.2017.03.056 28442313

[14] Fattori E, Cappelletti M, Costa P, Sellitto C, Cantoni L, Carelli M, et al. Defective inflammatory response in interleukin 6-deficient mice. J Exp Med. 1994;180: 1243–1250. doi: 10.1084/jem.180.4.1243 7931061 PMC2191674

[15] Tremblay L, Valenza F, Ribeiro SP, Li J, Slutsky AS. Injurious ventilatory strategies increase cytokines and c-fos m-RNA expression in an isolated rat lung model. J Clin Invest. 1997;99: 944–952. doi: 10.1172/JCI119259 9062352 PMC507902

[16] Tsujimoto H, Ono S, Mochizuki H, Aosasa S, Majima T, Ueno C, et al. Role of macrophage inflammatory protein 2 in acute lung injury in murine peritonitis. J Surg Res. 2002;103: 61–67. doi: 10.1006/jsre.2001.6325 11855919

[17] Xu WB, Haddad EB, Tsukagoshi H, Adcock I, Barnes PJ, Chung KF. Induction of macrophage inflammatory protein 2 gene expression by interleukin 1 beta in rat lung. Thorax. 1995;50: 1136–1140. doi: 10.1136/thx.50.11.1136 8553267 PMC475083

[18] Warner RL, Paine R, Christensen PJ, Marletta MA, Richards MK, Wilcoxen SE, et al. Lung sources and cytokine requirements for in vivo expression of inducible nitric oxide synthase. Am J Respir Cell Mol Biol. 1995;12: 649–661. doi: 10.1165/ajrcmb.12.6.7539274 7539274

[19] Gómez MI, Prince A. Airway epithelial cell signaling in response to bacterial pathogens. Pediatr Pulmonol. 2008;43: 11–19. doi: 10.1002/ppul.20735 18041080

[20] Eisenhut M. The regulation of respiratory epithelial cell apoptosis by nitric oxide in acute lung injury. Pediatric Critical Care Medicine. 2007;8: 410. doi: 10.1097/01.PCC.0000269377.01454.D2 17622931

[21] Asadullah K, Sterry W, Volk HD. Interleukin-10 therapy—review of a new approach. Pharmacol Rev. 2003;55: 241–269. doi: 10.1124/pr.55.2.4 12773629

[22] Kung TT, Crawley Y, Luo B, Young S, Kreutner W, Chapman RW. Inhibition of pulmonary eosinophilia and airway hyperresponsiveness in allergic mice by rolipram: involvement of endogenously released corticosterone and catecholamines. Br J Pharmacol. 2000;130: 457–463. doi: 10.1038/sj.bjp.0703308 10807686 PMC1572069

[23] Moore KW, O’Garra A, de Waal Malefyt R, Vieira P, Mosmann TR. Interleukin-10. Annu Rev Immunol. 1993;11: 165–190. doi: 10.1146/annurev.iy.11.040193.001121 8386517

[24] Fiorentino DF, Bond MW, Mosmann TR. Two types of mouse T helper cell. IV. Th2 clones secrete a factor that inhibits cytokine production by Th1 clones. J Exp Med. 1989;170: 2081–2095. doi: 10.1084/jem.170.6.2081 2531194 PMC2189521

[25] Lesur O, Langevin S, Berthiaume Y, Légaré M, Skrobik Y, Bellemare J-F, et al. Outcome value of Clara cell protein in serum of patients with acute respiratory distress syndrome. Intensive Care Med. 2006;32: 1167–1174. doi: 10.1007/s00134-006-0235-1 16794838

[26] Broeckaert F, Clippe A, Knoops B, Hermans C, Bernard A. Clara cell secretory protein (CC16): features as a peripheral lung biomarker. Ann N Y Acad Sci. 2000;923: 68–77. doi: 10.1111/j.1749-6632.2000.tb05520.x 11193780

[27] Kropski JA, Fremont RD, Calfee CS, Ware LB. Clara cell protein (CC16), a marker of lung epithelial injury, is decreased in plasma and pulmonary edema fluid from patients with acute lung injury. Chest. 2009;135: 1440–1447. doi: 10.1378/chest.08-2465 19188556 PMC2716712

[28] Fernandez-Bustamante A, Klawitter J, Repine JE, Agazio A, Janocha AJ, Shah C, et al. Early effect of tidal volume on lung injury biomarkers in surgical patients with healthy lungs. Anesthesiology. 2014;121: 469–481. doi: 10.1097/ALN.0000000000000301 24809976 PMC4165799

[29] Rava M, Tares L, Lavi I, Barreiro E, Zock J-P, Ferrer A, et al. Serum levels of Clara cell secretory protein, asthma, and lung function in the adult general population. J Allergy Clin Immunol. 2013;132: 230–232. doi: 10.1016/j.jaci.2013.01.023 23473837

[30] Crouch EC. Collectins and pulmonary host defense. Am J Respir Cell Mol Biol. 1998;19: 177–201. doi: 10.1165/ajrcmb.19.2.140 9698590

[31] Serpa Neto A, Campos PPZA, Hemmes SNT, Bos LD, Bluth T, Ferner M, et al. Kinetics of plasma biomarkers of inflammation and lung injury in surgical patients with or without postoperative pulmonary complications. Eur J Anaesthesiol. 2017;34: 229–238. doi: 10.1097/EJA.0000000000000614 28187051 PMC6696995

[32] Sunil VR, Vayas KN, Cervelli JA, Ebramova EV, Gow AJ, Goedken M, et al. Protective Role of Surfactant Protein-D Against Lung Injury and Oxidative Stress Induced by Nitrogen Mustard. Toxicol Sci. 2018;166: 108–122. doi: 10.1093/toxsci/kfy188 30060251 PMC6204765

[33] Shirasawa M, Fujiwara N, Hirabayashi S, Ohno H, Iida J, Makita K, et al. Receptor for advanced glycation end-products is a marker of type I lung alveolar cells. Genes Cells. 2004;9: 165–174. doi: 10.1111/j.1356-9597.2004.00712.x 15009093

[34] Determann RM, Millo JL, Waddy S, Lutter R, Garrard CS, Schultz MJ. Plasma CC16 levels are associated with development of ALI/ARDS in patients with ventilator-associated pneumonia: a retrospective observational study. BMC Pulm Med. 2009;9: 49. doi: 10.1186/1471-2466-9-49 19958527 PMC2794841

[35] Blondonnet R, Constantin J-M, Sapin V, Jabaudon M. A Pathophysiologic Approach to Biomarkers in Acute Respiratory Distress Syndrome. Dis Markers. 2016;2016: 3501373. doi: 10.1155/2016/3501373 26980924 PMC4766331

[36] Coxson HO, Dirksen A, Edwards LD, Yates JC, Agusti A, Bakke P, et al. The presence and progression of emphysema in COPD as determined by CT scanning and biomarker expression: a prospective analysis from the ECLIPSE study. Lancet Respir Med. 2013;1: 129–136. doi: 10.1016/S2213-2600(13)70006-7 24429093

[37] Stark JM, Coquet JM, Tibbitt CA. The Role of PPAR-γ in Allergic Disease. Curr Allergy Asthma Rep. 2021;21: 45. doi: 10.1007/s11882-021-01022-x 34697644 PMC8545719

[38] Birrell MA, Patel HJ, McCluskie K, Wong S, Leonard T, Yacoub MH, et al. PPAR-gamma agonists as therapy for diseases involving airway neutrophilia. Eur Respir J. 2004;24: 18–23. doi: 10.1183/09031936.04.00098303 15293600

[39] Kobayashi M, Thomassen MJ, Rambasek T, Bonfield TL, Raychaudhuri B, Malur A, et al. An inverse relationship between peroxisome proliferator-activated receptor gamma and allergic airway inflammation in an allergen challenge model. Ann Allergy Asthma Immunol. 2005;95: 468–473. doi: 10.1016/S1081-1206(10)61173-8 16312170

[40] Yin Y, Hou G, Li E, Wang Q, Kang J. PPARγ agonists regulate tobacco smoke-induced Toll like receptor 4 expression in alveolar macrophages. Respir Res. 2014;15: 28. doi: 10.1186/1465-9921-15-28 24612634 PMC4007599

[41] Remels AH, Gosker HR, Schrauwen P, Langen RC, Schols AM. Peroxisome proliferator-activated receptors: a therapeutic target in COPD? European Respiratory Journal. 2008;31: 502–508. doi: 10.1183/09031936.00068207 18310397

[42] Kytikova OYu, Perelman JM, Novgorodtseva TP, Denisenko YK, Kolosov VP, Antonyuk MV, et al. Peroxisome Proliferator-Activated Receptors as a Therapeutic Target in Asthma. PPAR Res. 2020;2020: 8906968. doi: 10.1155/2020/8906968 32395125 PMC7201810

[43] Fujii U, Miyahara N, Taniguchi A, Oda N, Morichika D, Murakami E, et al. Effect of a retinoid X receptor partial agonist on airway inflammation and hyperresponsiveness in a murine model of asthma. Respiratory Research. 2017;18: 23. doi: 10.1186/s12931-017-0507-z 28114934 PMC5260083

[44] Morichika D, Miyahara N, Fujii U, Taniguchi A, Oda N, Senoo S, et al. A retinoid X receptor partial agonist attenuates pulmonary emphysema and airway inflammation. Respir Res. 2019;20: 2. doi: 10.1186/s12931-018-0963-0 30606200 PMC6318915

[45] Lea S, Plumb J, Metcalfe H, Spicer D, Woodman P, Fox JC, et al. The effect of peroxisome proliferator-activated receptor-γ ligands on in vitro and in vivo models of COPD. European Respiratory Journal. 2014;43: 409–420. doi: 10.1183/09031936.00187812 23794466

[46] Stolk J, Stockley RA, Stoel BC, Cooper BG, Piitulainen E, Seersholm N, et al. Randomised controlled trial for emphysema with a selective agonist of the γ-type retinoic acid receptor. European Respiratory Journal. 2012;40: 306–312. doi: 10.1183/09031936.00161911 22282548

[47] Ferreira AE, Sisti F, Sônego F, Wang S, Filgueiras L, Brandt S, et al. PPAR-γ/IL-10 axis inhibits MyD88 expression and ameliorates murine polymicrobial sepsis. J Immunol. 2014;192: 2357–2365. doi: 10.4049/jimmunol.1302375 24489087 PMC3943997

[48] Chang T-H, Szabo E. Enhanced growth inhibition by combination differentiation therapy with ligands of peroxisome proliferator-activated receptor-gamma and inhibitors of histone deacetylase in adenocarcinoma of the lung. Clin Cancer Res. 2002;8: 1206–1212. 11948134

[49] Corona JC, Duchen MR. PPARγ as a therapeutic target to rescue mitochondrial function in neurological disease. Free Radic Biol Med. 2016;100: 153–163. doi: 10.1016/j.freeradbiomed.2016.06.023 27352979 PMC5145801

[50] Jamwal S, Blackburn JK, Elsworth JD. PPARγ/PGC1α signaling as a potential therapeutic target for mitochondrial biogenesis in neurodegenerative disorders. Pharmacol Ther. 2021;219: 107705. doi: 10.1016/j.pharmthera.2020.107705 33039420 PMC7887032

[51] Liu C, Tate T, Batourina E, Truschel ST, Potter S, Adam M, et al. Pparg promotes differentiation and regulates mitochondrial gene expression in bladder epithelial cells. Nat Commun. 2019;10: 4589. doi: 10.1038/s41467-019-12332-0 31597917 PMC6785552

[52] Fan W, Evans R. PPARs and ERRs: molecular mediators of mitochondrial metabolism. Curr Opin Cell Biol. 2015;33: 49–54. doi: 10.1016/j.ceb.2014.11.002 25486445 PMC4380823

[53] Wenz T, Wang X, Marini M, Moraes CT. A metabolic shift induced by a PPAR panagonist markedly reduces the effects of pathogenic mitochondrial tRNA mutations. J Cell Mol Med. 2011;15: 2317–2325. doi: 10.1111/j.1582-4934.2010.01223.x 21129152 PMC3361135

[54] Lee W, Lee CH, Lee J, Jeong Y, Park J-H, Nam I-J, et al. Botanical formulation, TADIOS, alleviates lipopolysaccharide (LPS)-Induced acute lung injury in mice via modulation of the Nrf2-HO-1 signaling pathway. J Ethnopharmacol. 2021;270: 113795. doi: 10.1016/j.jep.2021.113795 33421604 PMC7832766

[55] Lee W, Jeong SY, Gu MJ, Lim JS, Park EK, Baek M-C, et al. Inhibitory effects of compounds isolated from Dioscorea batatas Decne peel on particulate matter-induced pulmonary injury in mice. J Toxicol Environ Health A. 2019;82: 727–740. doi: 10.1080/15287394.2019.1646174 31342870

[56] Ma J, Kang SY, Meng X, Kang AN, Park JH, Park Y-K, et al. Effects of Rhizome Extract of Dioscorea batatas and Its Active Compound, Allantoin, on the Regulation of Myoblast Differentiation and Mitochondrial Biogenesis in C2C12 Myotubes. Molecules. 2018;23: 2023. doi: 10.3390/molecules23082023 30104552 PMC6222821

[57] Baek EB, Rho J-H, Jung E, Seo C-S, Kim J-H, Kwun H-J. Protective effect of Palmijihwanghwan in a mouse model of cigarette smoke and lipopolysaccharide-induced chronic obstructive pulmonary disease. BMC Complement Med Ther. 2021;21: 281. doi: 10.1186/s12906-021-03453-5 34784929 PMC8594196

[58] Ha H-J, Kim Y-J, Kweon K-T, Kim J-J. Review of the domestic research trends in the study of Korean herbal medicine with anti-inflammation effects. The Korea Journal of Herbology. 2011;26: 15–22. doi: 10.6116/KJH.2011.26.4.015

[59] Liu L, Xiong H, Ping J, Ju Y, Zhang X. Taraxacum officinale protects against lipopolysaccharide-induced acute lung injury in mice. J Ethnopharmacol. 2010;130: 392–397. doi: 10.1016/j.jep.2010.05.029 20510343

[60] Awortwe C, Sackeyfio AC, Osei-Safo D, Bugyei KA, Asiedu-Gyekye IJ. Dual effect of Taraxacum officinale leaves: Anticholinergic and inhibitory effect on inflammatory cells in ovalbumin-sensitized guinea-pigs. AJPP. 2011;5: 2613–2619. doi: 10.5897/AJPP11.616

[61] Do MH, Choi J, Kim Y, Park H-Y, Park Y, Ha SK, et al. Schizonepeta tenuifolia reduces methylglyoxal-induced cytotoxicity and oxidative stress in mesangial cells. Journal of Functional Foods. 2019;62: 103531. doi: 10.1016/j.jff.2019.103531

[62] Fung D, Lau CBS. Schizonepeta tenuifolia: chemistry, pharmacology, and clinical applications. J Clin Pharmacol. 2002;42: 30–36. doi: 10.1177/0091270002042001003 11817365

[63] Byun M-W. Schizonepeta tenuifolia ethanol extract exerts anti-inflammatory activity through the inhibition of TLR4 signaling in lipopolysaccharide-stimulated macrophage cells. J Med Food. 2014;17: 350–356. doi: 10.1089/jmf.2013.2928 24650252

[64] Lee W, Jeong Y, Park J-H, Lee CH, Yun N, Lee DS, et al. Water-Soluble Extract from Actinidia arguta (Siebold & Zucc.) Planch. ex Miq. and Perilla frutescens (L.) Britton, ACTPER, Ameliorates a Dry Skin-Induced Itch in a Mice Model and Promotes Filaggrin Expression by Activating the AhR Signaling in HaCaT Cells. Nutrients. 2019;11: 1366. doi: 10.3390/nu11061366 31216667 PMC6627490

[65] Lee W, Ko KR, Kim H, Lee DS, Nam I-J, Lim S, et al. Dehydrodiconiferyl Alcohol Inhibits Osteoclast Differentiation and Ovariectomy-Induced Bone Loss through Acting as an Estrogen Receptor Agonist. J Nat Prod. 2018;81: 1343–1356. doi: 10.1021/acs.jnatprod.7b00927 29869503

[66] Hammad DR, Elgazzar AG, Essawy TS, Abd El Sameie SA. Evaluation of serum interleukin-1 beta as an inflammatory marker in COPD patients. Egyptian Journal of Chest Diseases and Tuberculosis. 2015;64: 347–352. doi: 10.1016/j.ejcdt.2015.01.005

[67] Bhowmik A, Seemungal TA, Sapsford RJ, Wedzicha JA. Relation of sputum inflammatory markers to symptoms and lung function changes in COPD exacerbations. Thorax. 2000;55: 114–120. doi: 10.1136/thorax.55.2.114 10639527 PMC1745686

[68] Churg A, Dai J, Tai H, Xie C, Wright JL. Tumor necrosis factor-alpha is central to acute cigarette smoke-induced inflammation and connective tissue breakdown. Am J Respir Crit Care Med. 2002;166: 849–854. doi: 10.1164/rccm.200202-097OC 12231496

[69] Karadag F, Karul AB, Cildag O, Yilmaz M, Ozcan H. Biomarkers of systemic inflammation in stable and exacerbation phases of COPD. Lung. 2008;186: 403–409. doi: 10.1007/s00408-008-9106-6 18807087

[70] Prakash YS, Pabelick CM, Sieck GC. Mitochondrial Dysfunction in Airway Disease. Chest. 2017;152: 618–626. doi: 10.1016/j.chest.2017.03.020 28336486 PMC5812762

[71] Karim L, Lin C-R, Kosmider B, Criner G, Marchetti N, Bolla S, et al. Mitochondrial Ribosome Dysfunction in Human Alveolar Type II Cells in Emphysema. Biomedicines. 2022;10: 1497. doi: 10.3390/biomedicines10071497 35884802 PMC9313339

[72] van der Vliet A, Janssen-Heininger YMW, Anathy V. Oxidative stress in chronic lung disease: From mitochondrial dysfunction to dysregulated redox signaling. Mol Aspects Med. 2018;63: 59–69. doi: 10.1016/j.mam.2018.08.001 30098327 PMC6181583

[73] Kinnula VL, Fattman CL, Tan RJ, Oury TD. Oxidative stress in pulmonary fibrosis: a possible role for redox modulatory therapy. Am J Respir Crit Care Med. 2005;172: 417–422. doi: 10.1164/rccm.200501-017PP 15894605 PMC2718525

[74] Kinnula VL, Crapo JD. Superoxide dismutases in the lung and human lung diseases. Am J Respir Crit Care Med. 2003;167: 1600–1619. doi: 10.1164/rccm.200212-1479SO 12796054

[75] Powis G, Mustacich D, Coon A. The role of the redox protein thioredoxin in cell growth and cancer. Free Radic Biol Med. 2000;29: 312–322. doi: 10.1016/s0891-5849(00)00313-0 11035260

[76] Fattman CL, Schaefer LM, Oury TD. Extracellular superoxide dismutase in biology and medicine. Free Radic Biol Med. 2003;35: 236–256. doi: 10.1016/s0891-5849(03)00275-2 12885586

[77] Bargagli E, Olivieri C, Bennett D, Prasse A, Muller-Quernheim J, Rottoli P. Oxidative stress in the pathogenesis of diffuse lung diseases: a review. Respir Med. 2009;103: 1245–1256. doi: 10.1016/j.rmed.2009.04.014 19464864

[78] Hecker L. Mechanisms and consequences of oxidative stress in lung disease: therapeutic implications for an aging populace. Am J Physiol Lung Cell Mol Physiol. 2018;314: L642–L653. doi: 10.1152/ajplung.00275.2017 29351446 PMC5966777

[79] Rahman I, Kilty I. Antioxidant therapeutic targets in COPD. Curr Drug Targets. 2006;7: 707–720. doi: 10.2174/138945006777435254 16787173

[80] Hiben MG, de Haan L, Spenkelink B, Wesseling S, Vervoort J, Rietjens IMCM. Induction of peroxisome proliferator activated receptor γ (PPARγ) mediated gene expression and inhibition of induced nitric oxide production by Maerua subcordata (Gilg) DeWolf. BMC Complement Med Ther. 2020;20: 80. doi: 10.1186/s12906-020-2856-2 32164648 PMC7076844

[81] Rau O, Wurglics M, Paulke A, Zitzkowski J, Meindl N, Bock A, et al. Carnosic acid and carnosol, phenolic diterpene compounds of the labiate herbs rosemary and sage, are activators of the human peroxisome proliferator-activated receptor gamma. Planta Med. 2006;72: 881–887. doi: 10.1055/s-2006-946680 16858665

[82] Komeili Movahhed T, Moslehi A, Golchoob M, Ababzadeh S. Allantoin improves methionine-choline deficient diet-induced nonalcoholic steatohepatitis in mice through involvement in endoplasmic reticulum stress and hepatocytes apoptosis-related genes expressions. Iran J Basic Med Sci. 2019;22: 736–744. doi: 10.22038/ijbms.2019.33553.8012 32373294 PMC7196357

[83] Lee M-Y, Lee N-H, Jung D, Lee J-A, Seo C-S, Lee H, et al. Protective effects of allantoin against ovalbumin (OVA)-induced lung inflammation in a murine model of asthma. Int Immunopharmacol. 2010;10: 474–480. doi: 10.1016/j.intimp.2010.01.008 20100599

[84] Hamidi-zad Z, Moslehi A, Rastegarpanah M. Attenuating effects of allantoin on oxidative stress in a mouse model of nonalcoholic steatohepatitis: role of SIRT1/Nrf2 pathway. Res Pharm Sci. 2021;16: 651–659. doi: 10.4103/1735-5362.327511 34760013 PMC8562413

[85] Yan Z, Huang Q-L, Chen J, Liu F, Wei Y, Chen S-L, et al. Chicoric acid alleviates LPS-induced inflammatory response through miR-130a-3p/IGF-1pathway in human lung A549 epithelial cells. Eur J Inflamm. 2021;19: 20587392211038244. doi: 10.1177/20587392211038244

[86] Lee NY, Chung K-S, Jin JS, Bang KS, Eom Y-J, Hong C-H, et al. Effect of Chicoric Acid on Mast Cell-Mediated Allergic Inflammation in Vitro and in Vivo. J Nat Prod. 2015;78: 2956–2962. doi: 10.1021/acs.jnatprod.5b00668 26593037

[87] Ding H, Ci X, Cheng H, Yu Q, Li D. Chicoric acid alleviates lipopolysaccharide-induced acute lung injury in mice through anti-inflammatory and anti-oxidant activities. Int Immunopharmacol. 2019;66: 169–176. doi: 10.1016/j.intimp.2018.10.042 30466029

[88] Liu Q, Fang J, Chen P, Die Y, Wang J, Liu Z, et al. Chicoric acid improves neuron survival against inflammation by promoting mitochondrial function and energy metabolism. Food Funct. 2019;10: 6157–6169. doi: 10.1039/c9fo01417a 31501849

[89] Hussein OE, Hozayen WG, Bin-Jumah MN, Germoush MO, Abd El-Twab SM, Mahmoud AM. Chicoric acid prevents methotrexate hepatotoxicity via attenuation of oxidative stress and inflammation and up-regulation of PPARγ and Nrf2/HO-1 signaling. Environ Sci Pollut Res Int. 2020;27: 20725–20735. doi: 10.1007/s11356-020-08557-y 32246423

[90] Taheri Y, Sharifi-Rad J, Antika G, Yılmaz YB, Tumer TB, Abuhamdah S, et al. Paving Luteolin Therapeutic Potentialities and Agro-Food-Pharma Applications: Emphasis on In Vivo Pharmacological Effects and Bioavailability Traits. Oxid Med Cell Longev. 2021;2021: 1987588. doi: 10.1155/2021/1987588 34594472 PMC8478534

[91] Kure A, Nakagawa K, Kondo M, Kato S, Kimura F, Watanabe A, et al. Metabolic Fate of Luteolin in Rats: Its Relationship to Anti-inflammatory Effect. J Agric Food Chem. 2016;64: 4246–4254. doi: 10.1021/acs.jafc.6b00964 27170112

[92] Park CM, Song Y-S. Luteolin and luteolin-7-O-glucoside inhibit lipopolysaccharide-induced inflammatory responses through modulation of NF-κB/AP-1/PI3K-Akt signaling cascades in RAW 264.7 cells. Nutr Res Pract. 2013;7: 423–429. doi: 10.4162/nrp.2013.7.6.423 24353826 PMC3865263

[93] Yue L, Yao H. Mitochondrial dysfunction in inflammatory responses and cellular senescence: pathogenesis and pharmacological targets for chronic lung diseases. Br J Pharmacol. 2016;173: 2305–2318. doi: 10.1111/bph.13518 27189175 PMC4945771

[94] Michaeloudes C, Bhavsar PK, Mumby S, Chung KF, Adcock IM. Dealing with Stress: Defective Metabolic Adaptation in Chronic Obstructive Pulmonary Disease Pathogenesis. Ann Am Thorac Soc. 2017;14: S374–S382. doi: 10.1513/AnnalsATS.201702-153AW 29161091 PMC5711272

[95] Cloonan SM, Choi AMK. Mitochondria in lung disease. J Clin Invest. 126: 809–820. doi: 10.1172/JCI81113 26928034 PMC4767339

[96] Caldeira D de AF, Weiss DJ, Rocco PRM, Silva PL, Cruz FF. Mitochondria in Focus: From Function to Therapeutic Strategies in Chronic Lung Diseases. Frontiers in Immunology. 2021;12. Available: https://www.frontiersin.org/journals/immunology/articles/10.3389/fimmu.2021.782074 34887870 10.3389/fimmu.2021.782074 PMC8649841

[97] Rémy G, Grandjean T, Kervoaze G, Pichavant M, Chamaillard M, Gosset P. Implication of interleukin-10 in the development of COPD induced by cigarette smoke exposure in mice. European Respiratory Journal. 2013;42. Available: https://erj.ersjournals.com/content/42/Suppl_57/P614

[98] Sun Z, Chen A, Fang H, Sun D, Huang M, Cheng E, et al. B cell-derived IL-10 promotes the resolution of lipopolysaccharide-induced acute lung injury. Cell Death Dis. 2023;14: 1–13. doi: 10.1038/s41419-023-05954-2 37443161 PMC10345008

[99] Schwab AD, Wyatt TA, Nelson AJ, Gleason A, Gaurav R, Romberger DJ, et al. Lung-delivered IL-10 therapy elicits beneficial effects via immune modulation in organic dust exposure-induced lung inflammation. Journal of Immunotoxicology. 2024;21: 2332172. doi: 10.1080/1547691X.2024.2332172 38563602 PMC11137733

[100] Silva BSA, Lira FS, Ramos D, Uzeloto JS, Rossi FE, Freire APCF, et al. Severity of COPD and its relationship with IL-10. Cytokine. 2018;106: 95–100. doi: 10.1016/j.cyto.2017.10.018 29108795

[101] Jiang S, Shan F, Zhang Y, Jiang L, Cheng Z. Increased serum IL-17 and decreased serum IL-10 and IL-35 levels correlate with the progression of COPD. Int J Chron Obstruct Pulmon Dis. 2018;13: 2483–2494. doi: 10.2147/COPD.S167192 30154651 PMC6108328

[102] Rojas-Quintero J, Laucho-Contreras ME, Wang X, Fucci Q-A, Burkett PR, Kim S-J, et al. CC16 augmentation reduces exaggerated COPD-like disease in Cc16-deficient mice. JCI Insight. 2023;8: e130771. doi: 10.1172/jci.insight.130771 36787195 PMC10070105

[103] Yoon JM, Lee K-H, Lee SM, Lim J-J, Yang S-C, Yoo C-G, et al. The immune modulation of Clara cell-10 in human peripheral monocytes and dendritic cells. Int J Mol Med. 2010;26: 415–423. 20664959

[104] Zhai J, Emond MJ, Spangenberg A, Stern DA, Vasquez MM, Blue EE, et al. Club cell secretory protein and lung function in children with cystic fibrosis. J Cyst Fibros. 2022;21: 811–820. doi: 10.1016/j.jcf.2022.03.007 35367162 PMC9509401

[105] Mu W, Ouyang X, Agarwal A, Zhang L, Long DA, Cruz PE, et al. IL-10 suppresses chemokines, inflammation, and fibrosis in a model of chronic renal disease. J Am Soc Nephrol. 2005;16: 3651–3660. doi: 10.1681/ASN.2005030297 16251240

[106] Wang X, Wong K, Ouyang W, Rutz S. Targeting IL-10 Family Cytokines for the Treatment of Human Diseases. Cold Spring Harb Perspect Biol. 2019;11: a028548. doi: 10.1101/cshperspect.a028548 29038121 PMC6360861

[107] Millar AB. IL‐10: another therapeutic target in idiopathic pulmonary fibrosis? Thorax. 2006;61: 835–836. doi: 10.1136/thx.2006.060772 17008481 PMC2104749

[108] Cintra DE, Pauli JR, Araújo EP, Moraes JC, de Souza CT, Milanski M, et al. Interleukin-10 is a protective factor against diet-induced insulin resistance in liver. J Hepatol. 2008;48: 628–637. doi: 10.1016/j.jhep.2007.12.017 18267346

[109] Peng S-G, Pang Y-L, Zhu Q, Kang J-H, Liu M-X, Wang Z. Chlorogenic Acid Functions as a Novel Agonist of PPARγ2 during the Differentiation of Mouse 3T3-L1 Preadipocytes. Biomed Res Int. 2018;2018: 8594767. doi: 10.1155/2018/8594767 30627576 PMC6304673

[110] Lataliza AAB, de Assis PM, da Rocha Laurindo L, Gonçalves ECD, Raposo NRB, Dutra RC. Antidepressant-like effect of rosmarinic acid during LPS-induced neuroinflammatory model: The potential role of cannabinoid receptors/PPAR-γ signaling pathway. Phytother Res. 2021;35: 6974–6989. doi: 10.1002/ptr.7318 34709695

[111] Han J, Wang D, Ye L, Li P, Hao W, Chen X, et al. Rosmarinic Acid Protects against Inflammation and Cardiomyocyte Apoptosis during Myocardial Ischemia/Reperfusion Injury by Activating Peroxisome Proliferator-Activated Receptor Gamma. Front Pharmacol. 2017;8: 456. doi: 10.3389/fphar.2017.00456 28744220 PMC5504166

[112] Agrawal YO, Sharma PK, Shrivastava B, Ojha S, Upadhya HM, Arya DS, et al. Hesperidin produces cardioprotective activity via PPAR-γ pathway in ischemic heart disease model in diabetic rats. PLoS One. 2014;9: e111212. doi: 10.1371/journal.pone.0111212 25369053 PMC4219710

