# Supplementary material for: Botanical formulation HX110B ameliorates PPE-induced emphysema in mice via regulation of PPAR/RXR signaling pathway
Source: PLoS One. 2024 Jul 25;19(7):e0305911. doi: 10.1371/journal.pone.0305911 (PMC11271920; doi:10.1371/journal.pone.0305911)
Supplement: S1 Table — (DOCX) [file pone.0305911.s001.docx]

**S1 Table. Primer sequences**

| Name | | Sequence (5′ → 3′) |
| --- | --- | --- |
| GAPDH (mouse) | Forward | AGCCTCGTCCCGTAGACAA |
|  | Reverse | AATCTCCACTTTGCCACTGC |
| IL-6 (mouse) | Forward | TAGTCCTTCCTACCCCAATTTCC |
|  | Reverse | TTGGTCCTTAGCCACTCCTTC |
| IL-1β (mouse) | Forward | TGTGCAAGTGTCTGAAGCAGC |
|  | Reverse | TGGAAGCAGCCCTTCATCTT |
| MIP-2 (mouse) | Forward | CACCAACCACCAGGCTACA |
|  | Reverse | GCTTCAGGGTCAAGGCAAAC |
| iNOS (mouse) | Forward | CGAAACGCTTCACTTCCAA |
|  | Reverse | TGAGCCTATATTGCTGTGGCT |
| IL-10 (mouse) | Forward | CCCTGGGTGAGAAGCTGAAG |
|  | Reverse | CACTGCCTTGCTCTTATTTTCACA |
| CC16 (mouse) | Forward | ATGAAGATCGCCATCACAATCA |
|  | Reverse | GAATCTTAAATCTTGCTTACACAG |
| SP-D (mouse) | Forward | GCCTGGTCGTGATGGACGGG |
|  | Reverse | AGGGCCCTGCAACCCTGAGA |
| sRAGE (mouse) | Forward | ACTACCGAGTCCGAGTCTACC |
|  | Reverse | GTAGCTTCCCTCAGACACACA |
| ND1 (mouse) | Forward | ATTACTTCTGCCAGCCTGACC |
|  | Reverse | GGCCCGGTTTGTTTCTGCTA |
| NDUFB9 (mouse) | Forward | CTGGCAGAATCAGCATCCTCAG |
|  | Reverse | TGCTTTCTCAGAGGGATGCCAG |
| cytB (mouse) | Forward | GGCTACGTCCTTCCATGAGG |
|  | Reverse | TGGGATGGCTGATAGGAGGT |
| UQCRB (mouse) | Forward | CCATAAGAAGGCTTCCTGAGGAC |
|  | Reverse | TTTGTCCACTGATCCTTAGGCAAG |
| COX2 (mouse) | Forward | ACCTGGTGAACTACGACTGCT |
|  | Reverse | TCCTAGGGAGGGGACTGCTC |
| ATP5A1 (mouse) | Forward | TGGTGAAGAGACTGACGGATGC |
|  | Reverse | TCAAAGCGTGCTTGCCGTTGTC |
| GAPDH (human) | Forward | CCCCTTCATTGACCTCAACT |
|  | Reverse | ATGACCTTGCCCACAGCCTT |
| IL-10 (human) | Forward | TCTCCGAGATGCCTTCAGCAGA |
|  | Reverse | TCAGACAAGGCTTGGCAACCCA |
| CC16 (human) | Forward | CTTTCAGCGTGTCATCGAAA |
|  | Reverse | TGATGCTTTCTCTGGGCTTT |
| SP-D (human) | Forward | TGCTGCTCTTCCTCCTCTCTGC |
|  | Reverse | GGGCGTTGTTCTGTGGGAGTAG |
| sRAGE (human) | Forward | CTGATCCTCCCACAGAGCC |
|  | Reverse | CAGGACCAGGGAACCTACAG |
