# Supplementary material for: Botanical formulation HX110B ameliorates PPE-induced emphysema in mice via regulation of PPAR/RXR signaling pathway
Source: PLoS One. 2024 Jul 25;19(7):e0305911. doi: 10.1371/journal.pone.0305911 (PMC11271920; doi:10.1371/journal.pone.0305911)
Supplement: S1 Fig — (DOCX) [file pone.0305911.s002.docx]

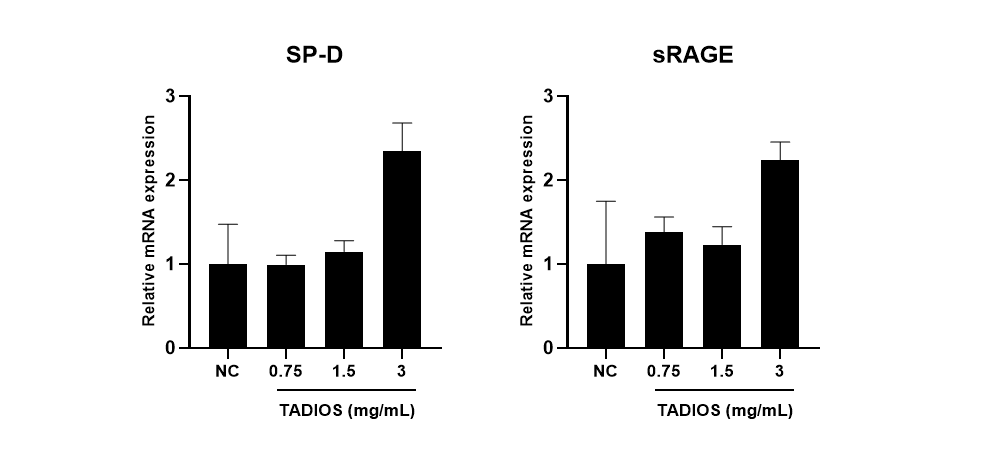


**S1 Fig.** **TADIOS does not significantly regulate the expression of SP-D and sRAGE in BEAS-2B cells.**

BEAS-2B cells were cultured with various concentrations of TADIOS (0.75, 1.5, and 3 mg/mL) for 48 h. (A and B) Changes in SP-D and sRAGE RNA levels were measured.
